# Haplotype-resolved chromosome-level genome assemblies of four *Diamesa* species reveal the genetic basis of cold tolerance and high-altitude adaptations in arctic chironomids

**DOI:** 10.1093/gigascience/giaf160

**Published:** 2025-12-22

**Authors:** Sarah L F Martin, Renato La Torre, Bram Danneels, Ave Tooming-Klunderud, Morten Skage, Spyridon Kollias, Ole Kristian Tørresen, Mohsen Falahati Anbaran, Elisabeth Stur, Kjetill S Jakobsen, Michael D Martin, Torbjørn Ekrem

**Affiliations:** Department of Natural History, NTNU University Museum, Norwegian University for Science and Technology, Trondheim 7491, Norway; Department of Natural History, NTNU University Museum, Norwegian University for Science and Technology, Trondheim 7491, Norway; Computational Biology Unit, Departme nt of Informatics, University of Bergen, Bergen 5020, Norway; Centre for Ecological and Evolutionary Synthesis, Department of Biosciences, University of Oslo, Oslo 0316, Norway; Centre for Ecological and Evolutionary Synthesis, Department of Biosciences, University of Oslo, Oslo 0316, Norway; Centre for Ecological and Evolutionary Synthesis, Department of Biosciences, University of Oslo, Oslo 0316, Norway; Centre for Ecological and Evolutionary Synthesis, Department of Biosciences, University of Oslo, Oslo 0316, Norway; Department of Natural History, NTNU University Museum, Norwegian University for Science and Technology, Trondheim 7491, Norway; Department of Natural History, NTNU University Museum, Norwegian University for Science and Technology, Trondheim 7491, Norway; Centre for Ecological and Evolutionary Synthesis, Department of Biosciences, University of Oslo, Oslo 0316, Norway; Department of Natural History, NTNU University Museum, Norwegian University for Science and Technology, Trondheim 7491, Norway; Department of Natural History, NTNU University Museum, Norwegian University for Science and Technology, Trondheim 7491, Norway

**Keywords:** Chironomidae, reference genomes, biodiversity genomics, adaptation, species delimitation, evolution

## Abstract

**Background:**

Arctic and alpine insects experience extreme environmental stressors, yet the genomic basis of their adaptation is poorly understood. *Diamesa* midges (Diptera: Chironomidae) are cold-adapted insects inhabiting glacial and high-altitude freshwater ecosystems, but no chromosome-level genomes have been available to date.

**Findings:**

We present the first haplotype-resolved, chromosome-level genomes for four *Diamesa* species (*Diamesa hyperborea, Diamesa lindrothi, Diamesa serratosioi*, and *Diamesa tonsa*), assembled using PacBio HiFi sequencing and Hi-C scaffolding. The assemblies show high completeness and *k*-mer representation. Phylogenomic analyses place Diamesinae as sister to other Chironomidae except Podonominae, and comparisons suggest introgression between the distinct species *D. hyperborea* and *D. tonsa*. Comparative genomic analyses across 20 Diptera species identified significant gene family contractions in *Diamesa* related to oxygen transport and metabolism, consistent with adaptation to high-altitude, low-oxygen environments. Expansions were observed in histone-related and Toll-like receptor gene families, suggesting roles in chromatin remodeling and immune regulation under cold stress. A glucose dehydrogenase gene family was significantly expanded across all cold-adapted species studied, implicating it in cryoprotectant synthesis and oxidative stress mitigation. *Diamesa* exhibited the largest gene family contraction at any phylogenetic node, with limited overlap in expansions with other cold-adapted Diptera, indicating lineage-specific adaptation.

**Conclusions:**

Our findings support the hypothesis that genome size condensation and selective gene family changes underpin survival in cold environments. These new genome assemblies provide a valuable resource for studying adaptation, speciation, and conservation in cold-specialist insects. Future integration of gene expression and population genomics will further clarify the evolutionary resilience of *Diamesa* in a warming world.

## Background

Understanding the effects of climate change on insect populations is of fundamental importance for the management and conservation of freshwater and terrestrial ecosystems [1]. While dispersal to habitable areas is a response option for lowland and temperate taxa, high-altitude or arctic species have nowhere to run to as temperatures and other climatic variables change to levels beyond those critical for their survival [2]. Remaining, then, is the species’ ability to adapt to the changing environment through altered behavior, change in phenology, or physical appearance [3]. The adaptive potential (i.e., the capacity to respond to changing selection pressures) of an insect is dependent on multiple factors, including genomic mechanisms [4]. Thus, our ability to understand an organism’s capacity to cope with environmental change should include knowledge of its genome, perhaps especially of nonmodel organisms with adaptations to extreme environments [5].

Midges of the family Chironomidae (Diptera) are among the most abundant and species-rich aquatic insects worldwide [6]. The family is represented in all continents and biogeographic regions, including the Antarctic mainland [7], and is one of few insect groups in which multiple evolutionary lineages have adapted to life in the marine environment [6]. While the immature stages of the majority of species are aquatic, terrestrial and semi-terrestrial species are also common [8]. Some species have adapted to life in extreme environments and are capable of enduring desiccation [9], heavy pollution [10], low pH [11], high salinity [12], and low temperatures [13]. Species of the subfamily Diamesinae typically have larvae and pupae associated with cold, flowing waters or nutrient-poor lakes [14], with the genus *Diamesa* colonizing such habitats mainly in the Northern Hemisphere and in some regions being valuable bioindicators of cold mountain waters [13]. A better understanding of the genomic architecture in *Diamesa* species could therefore help identify regions associated with adaptation to cold temperatures in these insects. Moreover, as the genus *Diamesa* has seen a rather recent radiation in the Neogene period [13, 14], with reported lower evolutionary rates of mitochondrial protein-coding genes [15] and suspected hybridization [16], it is of interest to explore the genomic divergence between closely related species.

Advances in long-read sequencing technologies, such as PacBio and Oxford Nanopore, in combination with Hi-C scaffolding, have made it possible to generate chromosome-level genome assemblies even for nonmodel organisms with small genomes, such as *Diamesa* species. These modern approaches provide vastly improved contiguity and completeness compared to earlier short-read assemblies, such as the genome of *Belgica antarctica* [17], which was highly fragmented and lacked chromosomal resolution. Importantly, global initiatives such as the Earth BioGenome Project [18] are accelerating the production of high-quality reference genomes across the tree of life, including ecologically important but historically understudied taxa. By providing standardized protocols and infrastructure, these efforts are enabling comprehensive biodiversity genomics and helping to close longstanding taxonomic and genomic gaps.

The genome of the Antarctic midge *B. antarctica* was the first chironomid genome to be published [17]. At the time, it was the smallest insect genome sequenced (99 Mbp), which was featured as a likely adaptation to an extreme environment. Sequenced genomes from other midges in Chironomidae as well as the sister family Ceratopogonidae indicate, however, that the small genome size is a plesiomorphic trait for the family [19]. Over the past decade, there have been published chromosome-level genomes of 10 species in four subfamilies: *Parochlus steinenii* Gercke, 1889 (Podonominae); *Clunio marinus* Haliday, 1855; *Smittia aterrima* (Meigen, 1818); *Smittia pratorum* (Goetghebuer, 1927) (Orthocladiinae); *Chironomus riparius* Meigen, 1804; *Chironomus tentans* Fabricius, 1805; *Polypedilum pembai* Cornette et al., 2017; *Polypedilum vanderplanki* Hinton, 1951; *Tanytarsus gracilentus* (Holmgren, 1883) (Chironominae); and *Propsilocerus akamusi* (Tokunaga, 1938) (Prodiamesinae) (Table 1). Thus, after the recent establishment of the Protanypodinae [14], there are eight subfamilies within the Chironomidae that lack published genomes. While some of the previously published studies on chironomid genomes are largely descriptive (e.g., [20, 21]), others discuss genomic mechanisms underlying tolerance to heavy metal exposure [10], anhydrobiosis [22], low temperatures [17], or generally stressful environments [19]. For the marine midge *Clunio marinus*, whose reproduction is timed with the tide, Kaiser et al. [23] used the genomes from five geographically different lineages to map loci for circalunar and circadian chronotypes. It is worth mentioning that closer examination of the COI barcodes from the populations used to generate the *Smittia* genomes [20] indicate that these do not belong to the species assigned in the publication but to other members of the same genus.

**Table 1:** Available whole genomes in Chironomidae

Species	Subfamily	Total length (Mbp)	BUSCO completeness (%)^a^	Accession number	Reference
*Parochlus steinenii*	Podonominae	143.57	98.9% [S:97.7%, D:1.2%], F:0.1%, M:1.0%	GCA_038,502,155.1	[19, 24]
*Belgica antarctica*	Orthocladiinae	89.58	98.2% [S:97.1%, D:1.2%], F:0.7%, M:1.1%	GCA_000,775,305.1	[17]
*Clunio marinus*	Orthocladiinae	85.49	98.7% [S:97.4%, D:1.2%], F:0.1%, M:1.2%	GCA_900,005,825.1	[23]
*Smittia aterrima* ^b^	Orthocladiinae	78.45	98.3% [S:96.3%, D:2.0%], F:0.1%, M:1.6%	GCA_033,063,855.1	[20]
*Smittia pratorum* ^b^	Orthocladiinae	71.56	97.5% [S:96.0%, D:1.5%], F:0.0%, M:2.5%	GCA_033,064,975.1	[20]
*Chironomus riparius*	Chironominae	191.84	99.0% [S:96.7%, D:2.3%], F:0.0%, M:1.0%	GCA_917,627,325.3	[21]
*Chironomus tentans*	Chironominae	213.46	97.7% [S:94.7%, D:3.0%], F:1.1%, M:1.2%	GCA_000,786,525.1	[25]
*Polypedilum pembai*	Chironominae	122.92	98.0% [S:95.4%, D:2.6%], F:0.1%, M:1.9%	GCA_014,622,435.1	[26]
*Polypedilum vanderplanki*	Chironominae	118.97	98.8% [S:96.6%, D:2.1%], F:0.1%,M:1.1%	GCA_018,290,095.1	[22]
*Tanytarsus gracilentus*	Chironominae	91.83	98.8% [S:97.8%, D:1.0%], F:0.0%, M:1.2%	GCA_038,502,055.1	[19]
*Propsilocerus akamusi*	Protanypodinae	85.84	98.8% [S:97.1%, D:1.7%], F:0.0%, M:1.2%	GCA_018,397,935.1	[27]

^a^Based on the insecta_odb10 dataset (1,366 genes). First percentage indicates complete BUSCO. ^b^Comparison of COI barcodes indicates that these genomes belong to other species in the genus *Smittia*. D: duplicated; F: fragmented; M: missing; S: single-copy.

Here, we generate high-quality haplotype-resolved chromosome-level assemblies for four species of *Diamesa* (subfamily Diamesinae), the first genomes generated for this subfamily using long-read sequencing (PacBio HiFi) and long-range chromosomal contact maps (Hi-C). We use these genomes to investigate the evolutionary mechanisms employed by these species to overcome extreme environments, with a particular focus on cold and high-altitude tolerance.

## Material and Methods

### Biological sample collection and identification

Fieldwork specifically for this project was conducted at 1,100 m elevation in the Rondane National Park, Norway, in July 2022, where previous studies had documented 12 different species of *Diamesa*. Adult specimens were caught by sweeping vegetation near the stream Vidjedalsbekken, and live specimens were individually identified to the genus level in the field by isolating them in 2-mL glass vials. One leg from each selected specimen was separated from the main body and preserved in ethanol, while the remaining body was snap-frozen in liquid nitrogen, labeled with consecutive numbers to ensure future association of body parts, and stored at −80°C until ready for DNA extraction.

**Figure 1:**
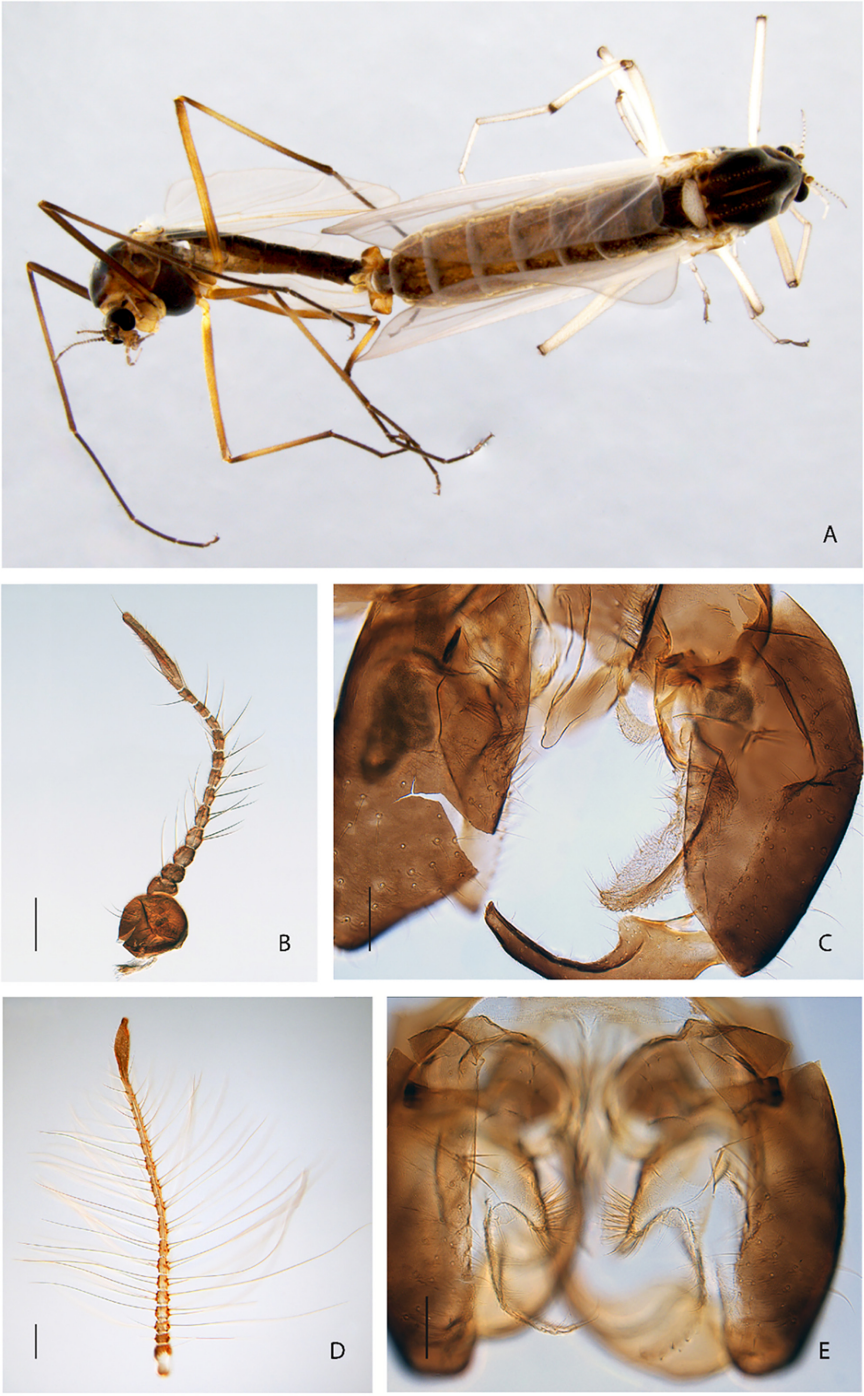
Morphological characteristics of *D. hyperborea* (A–C) and *D. tonsa* (D, E). (A) Male and female adult in copula. (B, D) Adult male antenna. (C, E) Male hypopygium. Scale bar in B and D = 100 μm, scale bar in C and E = 75 μm. Photos: Elisabeth Stur & Torbjørn Ekrem.

All *Diamesa* specimens were DNA barcoded using the following procedure. DNA from ethanol-preserved legs was extracted using the DNeasy Blood and Tissue kit (Qiagen) following the standard protocol, except elution was done twice with 50 µL buffer, with eluate from the first round used in the second round to increase the final DNA concentration. PCR of the cytochrome c oxidase subunit I (COI) barcode fragment was conducted using the primers LCO1490 and HCO2198 [28], along with the QIAGEN Multiplex PCR Kit. The temperature profile consisted of 95°C for 5 minutes for an initial denaturation, followed by 5 cycles of 94°C for 30 seconds, 45°C for 30 seconds, and 72°C for 60 seconds, as well as 35 cycles of 94°C for 30 seconds, 51°C for 30 seconds, and 72°C for 60 seconds, ending with a final extension of 72°C for 5 minutes. PCR products were cleaned using the ExoSAP-IT PCR Product Cleanup Reagent (Thermo Fisher Scientific) and sequenced in the reverse direction at Eurofins Genomics using Sanger sequencing and BigDye termination. Sequences were trimmed for uncertain base calls at each end and identified against the entries in the Barcode of Life Data Systems (BOLD [29]) with special reference to previous records from the same locality. All sequences and metadata are available in BOLD under the dataset “DS-DIANOR1 Norwegian Diamesa for genomics” [30]. Based on morphological identification (see Fig. 1) using available literature [31–33] and the results from DNA barcoding, adult males of *D. hyperborea* Holmgren, 1869 (NCBI:txid984905), *D. tonsa* Haliday, 1856 (NCBI:txid1751538), and *D. serratosioi* Willassen, 1985 (NCBI:txid2578515), as well as 1 female of *D. lindrothi* Goetghebuer (NCBI:txid3021724), 1931, were selected for individual high-molecular-weight (HMW) DNA extraction. Prior to placement of snap-frozen specimens in extraction buffer, wings, antennae, and hypopygium (e.g., Fig. 1) of each specimen were dissected off and mounted in Euparal on slides for permanent storage as vouchers in the NTNU University Museum Natural History Collections (NTNU-VM, Supplementary Table S1).

Additional specimens were required for Hi-C analyses (locations in Fig. 1). For this, we used individual male adults of each of the 4 selected species that had been collected in 2008 and 2014, preserved in 96% ethanol, and stored at 4–5°C (details in Supplementary Table S1). These individuals were collected at or near the field locality, except *D. tonsa*, which was collected at the Gaula River near Kvål in Melhus kommune, Trøndelag, Norway (Supplementary Table S1). After collection, specimens were stored at −80°C.

### DNA extraction

Prior to DNA extraction, the specimens were taken from −80°C for brief microscopic examination, in which genitalia were removed for voucher collection. HMW DNA was extracted using the MagAttract HMW DNA kit (Qiagen) following the manufacturer’s protocol. The individual specimens were manually homogenized using a sterile pestle in the ATL buffer. The final elution was in 50 µL Buffer AE.

### Library preparation and sequencing

Before PacBio library preparation, genomic DNA (gDNA) was purified an additional time using AMPure PB beads (1:1 ratio). For 2 of the species, short fragment removal was performed using 0.5× AMPure beads (*D. serratosioi*) and 35% diluted AMPure PB beads (*D. hyperborea*). For all 4 species, 16–25 ng DNA was sheared into an average fragment size of 10–15 kbp using g-TUBEs (Covaris). Libraries were prepared following the PacBio protocol for the low-input DNA Procedure & Checklist—Preparing HiFi SMRTbell Libraries from Ultra-Low DNA Input. Libraries were size-selected using 35% diluted AMPure PB beads and following the PacBio protocol “Using AMPure® PB Beads for Size-Selection.” Final libraries were pooled before sequencing on the PacBio Sequel IIe instrument (Pacific Biosciences). The libraries were sequenced on one 8-M SMRT cell using the Sequel II Binding Kit 2.2 and Sequencing Chemistry v2.0. To increase the amount of data for *D. lindrothi*, the library was sequenced on approximately 5% of a 25-M SMRT cell on the Revio instrument (also PacBio) using Revio polymerase and sequencing chemistry. PacBio library prep and sequencing were performed by the Norwegian Sequencing Centre (NSC) at the University of Oslo.

For all species, the whole organism was used for generating Hi-C data. Hi-C libraries were prepared using the Arima High Coverage Hi-C kit (Arima Genomics), following the manufacturer’s recommendations for low-input samples and the user guide for animal tissues (document no. A160162 v01). For 3 of the species, 1 individual stored in EtOH was used as input to Hi-C library prep, while for *D. lindrothi*, 2 individuals stored in EtOH were used. Final libraries were quantified using the Kapa Library quantification kit for Illumina (Roche) and pooled with other libraries before sequencing on the Illumina NovaSeq X with the 2 × 150-bp paired-end mode (Illumina) at the NSC.

### Genome assembly

A full list of relevant software tools and versions is presented in Supplementary Table S2. We assembled the species using a prerelease of the EBP-Nor genome assembly pipeline [34]. KMC [35] was used to count *k*-mers of size 32 in the PacBio HiFi reads, excluding *k*-mers occurring more than 10,000 times. GenomeScope [36] was run on the *k*-mer histogram output from KMC to estimate genome size, heterozygosity, and repetitiveness, while ploidy level was investigated using Smudgeplot [36]. HiFiAdapterFilt [37] was applied on the HiFi reads to remove possible remnant PacBio adapter sequences. The filtered HiFi reads were assembled using hifiasm [38] with Hi-C integration, resulting in a pair of haplotype-resolved assemblies, pseudo-haplotype 1 (hap1) and pseudo-haplotype 2 (hap2). Unique *k*-mers in each assembly/pseudo-haplotype were identified using meryl [39] and used to create 2 sets of Hi-C reads, one without any *k*-mers occurring uniquely in hap1 and the other without *k*-mers occurring uniquely in hap2. Hi-C reads filtered based on *k*-mers were aligned to each scaffolded assembly using BWA-MEM [40] with -5SPM options. The alignments were sorted based on name using SAMtools [41] before applying SAMtools fixmate to remove unmapped reads and secondary alignments and to add mate score, and SAMtools markdup to remove duplicates. The resulting BAM files were used to scaffold the 2 assemblies using YaHS [42] with default options. FCS-GX [43] was used to search and remove foreign contaminant sequences. The mitochondrion was assembled from PacBio HiFi reads using Oatk [44] and a minimum synmcer coverage threshold value of either 150 or 100. Similar to the assembly and annotation, all the genome evaluation tools were implemented following the EBP-Nor pipeline [45]. Merqury [39] was used to assess the completeness and quality of the genome assemblies by comparing to the *k*-mer content of the Hi-C reads. BUSCO [46] was used to assess the completeness of the genome assemblies by comparing against the expected single-copy orthologous gene content in the insecta_odb10 lineage. Gfastats [47] was used to output different assembly statistics of the assemblies. The assemblies were manually curated using PretextView and Rapid Curation 2.0. Chromosomes were identified by inspecting the Hi-C contact map in PretextView. BlobToolKit and BlobTools2 [48], in addition to blobtk, were used to visualize assembly statistics. To generate the Hi-C contact map image, the Hi-C reads were mapped to the assemblies using BWA-MEM [40] using the same approach as above, before PretextMap was used to create a contact map that was visualized using PretextSnapshot. The mitochondrial genome was recovered for all species.

### Genome annotation

We annotated the genome assemblies using a prerelease version of the EBP-Nor genome annotation pipeline [49]. First, AGAT [50] scripts agat_sp_keep_longest_isoform.pl and agat_sp_extract_sequences.pl were used on the fruit fly (*Drosophila melanogaster* Meigen, 1830) genome assembly (BDGP6.46 [GCA_000,001,215.4] from Ensembl) and annotation to generate 1 protein (the longest isoform) per gene. Miniprot [51] was used to align the proteins to the curated assemblies. UniProtKB/Swiss-Prot [52] release 2022_03 and the Arthropoda part of OrthoDB v11 [53] were also aligned separately to the assemblies. Red [54] was run via redmask [55] on the assemblies to mask repetitive areas. In addition, we ran Earl Grey [56] to annotate transposable elements. GALBA [51, 57–60] was run with the fruit fly proteins using the miniprot mode on the masked assemblies. The funannotate-runEVM.py script from Funannotate was used to run EvidenceModeler [61] on the alignments of the fruit fly proteins, UniProtKB/Swiss-Prot proteins, Arthropoda proteins, and the predicted genes from GALBA. The resulting predicted proteins were compared to the protein repeats that Funannotate distributes using DIAMOND blastp, and the predicted genes were filtered based on this comparison using AGAT. The filtered proteins were compared to the UniProtKB/Swiss-Prot release 2022_03 using DIAMOND [58] blastp to find gene names, and InterProScan was used to discover functional domains. AGAT’s agat_sp_manage_functional_annotation.pl script was used to attach the gene names and functional annotations to the predicted genes. EMBLmyGFF3 [62] was used to combine the fasta files and GFF3 files into an EMBL format for submission to the European Nucleotide Archive.

### Phylogenetic and comparative genomic analysis

A multispecies comparative analysis was performed using the proteomes from haplotype 1 of the four *Diamesa* species in addition to all the species in Table 1. Peptide sequences and annotations were downloaded from Nell et al. [19] for all species except *Diamesa* and *Smittia*, while *Smittia* data were taken from Fu et al. [20]. The comparative genomic workflow used was based on the methods from La Torre et al. [63]. Specifically, proteins from all chromosomes/scaffolds for each species respectively were used, including only 1 representative isoform per protein-coding gene in the analyses. Orthologous gene groups (orthogroups) were inferred using OrthoFinder version 2.5.5 [64], from which those single-copy genes shared between all species were aligned with MAFFT version 7.515 [65] and trimmed with trimAl version 1.2 [66]. The alignment was used as input for constructing a maximum likelihood (ML) phylogeny using RAxML version 8.2.12 [67] under the substitution model LG + I + G4 + F, as determined using ModelTest-NG version 0.1.7 [68]. Phylogenetic hierarchical orthogroups (HOGs) were identified in a second OrthoFinder run specifying the single-copy orthogroup ML tree, from which uninformative (genes restricted to a single species) and large (>100 genes) gene families were discarded. This species tree was used along with the filtered gene families as inputs for CAFE version 5 [69] to estimate the patterns of expansion or contraction of gene families. A summary and plots of gene family dynamics were obtained using CafePlotter version 0.2.0 [70].

A Gene Ontology (GO) enrichment analysis using topGO version 2.59.0 [71] was performed for each of the *Diamesa* species separately, for both significantly contracting and expanding families. A Fisher exact test with the algorithm *weight01* and a *nodeSize* parameter of 10 was used for estimating significance in the functional terms Biological Process (BP), Molecular Function (MF), and Cellular Component (CC).

## Results and Discussion

### Genome assembly and genome annotation

**Figure 2:**
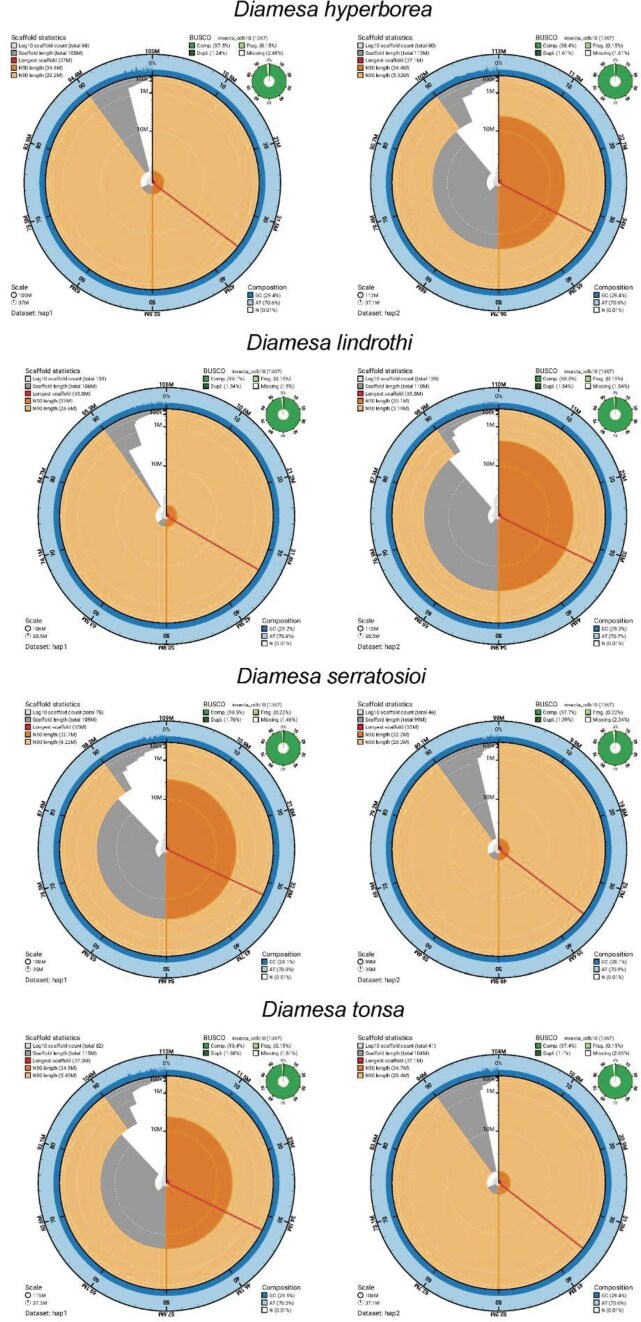
Metrics of the genome assemblies of four *Diamesa* species. The BlobToolKit Snailplots shows N50 metrics and BUSCO gene completeness. The two outermost bands of the circle signify GC versus AT composition at 0.1% intervals. Light orange shows the N90 scaffold length, while the deeper orange is N50 scaffold length. The red line shows the size of the largest scaffold. All the scaffolds are arranged in a clockwise manner from the largest to the smallest and are shown in darker gray with white lines at different orders of magnitude. The light gray shows the cumulative scaffold count. The scale inset in the lower left corner shows the total amount of sequence in the whole circle and the fraction of the circle encompassed in the largest scaffold. For every species, haplotype 1 is depicted on the left, and haplotype 2 is depicted on the right.

Here we present the first assembled genomes in the subfamily Diamesinae. Two haplotype-separated genomes could be assembled for all four *Diamesa* species investigated in this study. The final genome assemblies are between 98.9 and 115.1 Mbp in size, which is within the range of other Chironomidae genomes (Fig. 2, Tables 1 and 2).

**Table 2:** Assembly and annotation statistics for the four *Diamesa* genomes

Species	*D. hyperborea*		*D. lindrothi*		*D. serratosioi*		*D. tonsa*	
**Haplotype**	Hap1	Hap 2	Hap1	Hap 2	Hap1	Hap 2	Hap1	Hap 2
**Assembly span (Mbp)**	104.9	113.4	105.8	109.9	109.2	98.9	115.1	104.5
**Number of chromosomes identified**	3	3	4	4	3	3	3	3
**Number of contigs**	123	114	189	192	133	98	135	76
**Contig N_50_ length (Mbp)**	2.9	3.8	4.1	2.7	2.9	3.9	3.2	4.4
**Longest contig (Mbp)**	8.8	11.3	9.1	11.3	8.9	7.8	11.1	12.5
**Number of scaffolds**	66	60	139	139	75	46	82	41
**Scaffold N50 length (Mbp)**	34.6	34.4	33.0	33.1	32.7	32.2	34.5	34.7
**Longest scaffold (Mbp)**	36.9	37.1	35.5	35.5	35.0	34.9	37.2	37.1
**Genomic BUSCO completeness** **^a^**	C:97.5% [S:96.3%, D:1.2%], F:0.1%, M:2.3%	98.4% [S:96.8%, D:1.6%], F:0.1%, M:1.5%	98.1% [S:96.6%, D:1.5%], F:0.1%, M:1.8%	98.5% [S:96.9%, D:1.5%], F:0.1%, M:1.4%	98.5% [S:96.8%, D:1.8%], F:0.2%, M:1.2%	97.7% [S:96.3%, D:1.4%], F:0.2%, M:2.1%	98.4% [S:96.7%, D:1.7%], F:0.1%, M:1.5%	97.4% [S:96.3%, D:1.1%], F:0.1%, M:2.5%
**Number of annotated protein-coding genes**	10,798	11,097	10,638	10,831	11,113	10,472	11,375	10,535
**Number of protein-coding genes with functional domain** **^b^**	10,283	10,518	10,181	10,365	10,573	10,037	10,771	10,050
**Number of protein-coding genes with gene name**	6753	6842	6848	6887	6944	6714	6956	6680
**Annotation BUSCO completeness** **^a^**	C:94.9% [S:93.1%, D:1.8%], F:0.8%, M:4.3	95.1% [S:93.3%, D:1.8%], F:0.7%, M:4.2%	95.7% [S:94.1%, D:1.6%], F:0.6%, M:3.7%	96.1% [S:93.9%, D:2.2%], F:0.5%, M:3.4%	95.9% [S:94.1%, D:1.8%], F:0.5%, M:3.6%	95.1% [S:93.6%, D:1.5%], F:0.6%, M:4.3%	95.5% [S:93.7%, D:1.8%], F:0.6%, M:3.9%	94.8% [S:93.7%, D:1.1%], F:0.7%, M:4.5%
**HiFi read coverage**	62×		49×		60×		58×	
**Hi-C read coverage**	99×		114×		106×		97×	
**Consensus quality (QV) compared to HiFi (compared to Hi-C)**	64.3 (20.9)	63.5 (20.8)	59.1 (30.6)	59.9 (30.9)	63.1 (26.9)	64.6 (26.9)	63.1 (20.7)	66.7 (20.8)
**Consensus quality (QV) over both haplotypes**	63.9 (20.9)		59.5 (30.8)		63.7 (26.9)		64.5 (20.7)	
***K-*mer completeness (percentage; compared to HiFi reads)**	73.9	77.8	91.6	92.3	89.7	86.6	74.1	70.4
***K-*mer completeness over both haplotypes**	99.8		99.8		99.7		99.8	
**Estimated heterozygosity**	1.33%		0.48%		0.64%		1.73%	

a Based on the insecta_odb10 dataset (1,367 genes). First percentage indicates complete BUSCO. ^b^Number of genes annotated with a functional domain as found by InterProScan. D: duplicated; F: fragmented; M: missing; S: single-copy.

**Figure 3:**
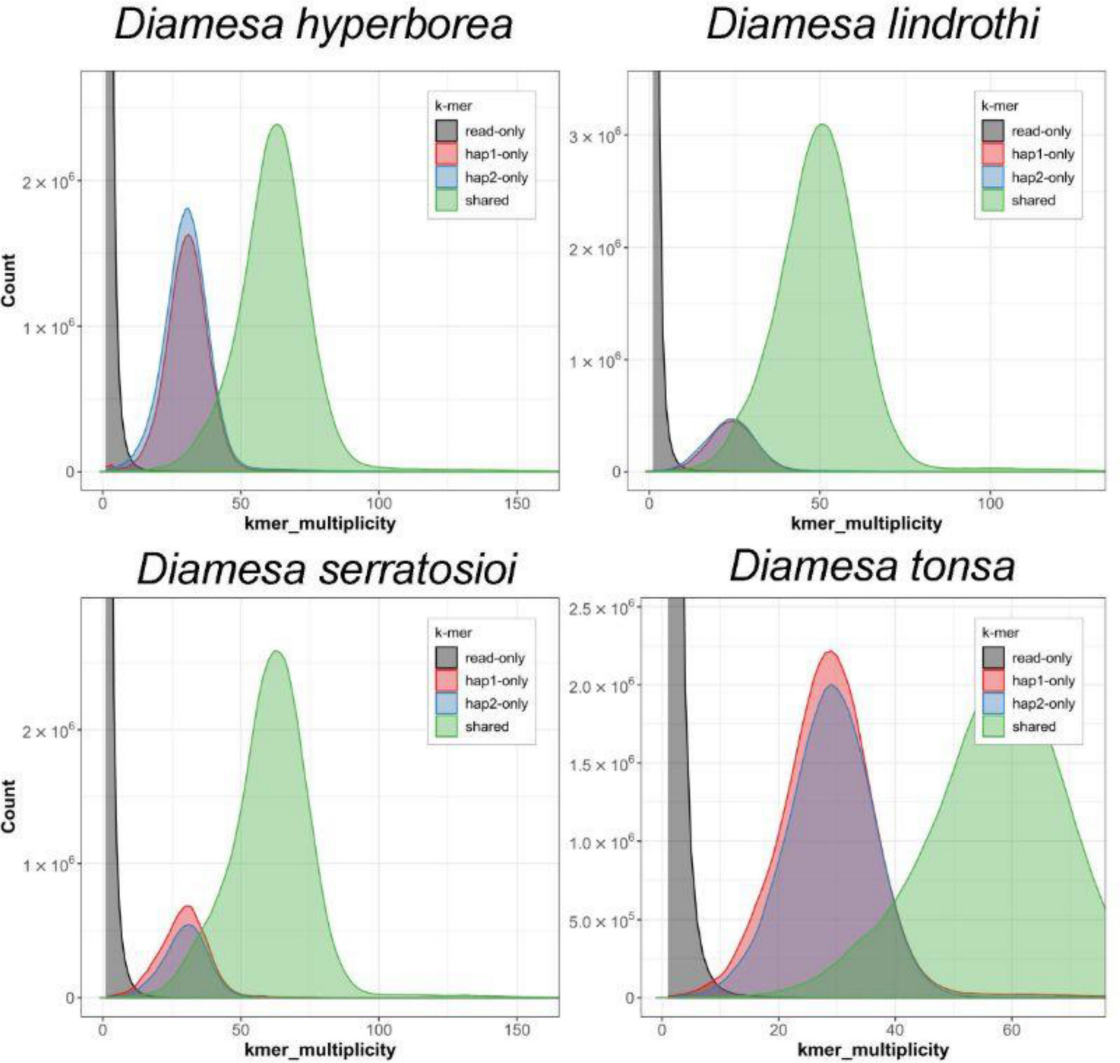
*K*-mer spectra of HiFi reads from the genomes of four *Diamesa* species. Distributions of *k*-mers found only in the reads (black), only in haplotype 1 (red), only in haplotype 2 (blue), and in both haplotypes (green). The x-axis depicts the number of unique *k*-mers, while the y-axis depicts the *k*-mer multiplicity (how often the *k*-mer is found in the set of HiFi reads).

The assembled genomes are slightly larger than their estimated genome size from the *k*-mer spectra (99.8 Mbp, 97.6 Mbp, 92.7 Mbp, and 99.1 Mbp predicted genome size for *D. hyperborea, D. lindrothi, D. serratosioi*, and *D. tonsa*, respectively). Contig and scaffold N_50_ values are generally high, ranging from 2.7 to 4.4 Mbp for contig N_50_ and 34.9 to 37.2 Mbp for scaffold N_50_. Three chromosomes were identified in *D. hyperborea, D. serratosioi*, and *D. tonsa*, while four chromosomes were identified in *D. lindrothi*. A scaffold of similar size was detected in the three other species but in each case was not labeled as a chromosome due to its presence in only one of the two haplotypes. The presence of four chromosomes is in line with karyotyping in other Diamesinae species [72]. The generated genomes have high BUSCO completeness (>97.4%) (Table 2 and Fig. 2), *k*-mer completeness (>99.7% over both haplotypes in all species) (Fig. 3), and consensus quality value (QV >59.1, where a QV of 50 corresponds to one error every 100,000 bp, or 99.999% accuracy). The *k*-mer spectra show a nice separation between *k*-mers derived from sequencing errors (low multiplicity, only found in reads), haplotype-specific *k*-mers (only found in one haplotype, multiplicity around half of the general coverage), and *k*-mers shared between both haplotypes (with a multiplicity similar to the coverage) (Fig. 3). Genome annotation of the assemblies identified between 10,472 and 11,375 protein-coding genes (Table 2). In most of the proteins (around 95%), at least one functional domain could be detected, and for a large part of the proteins (60–65%), a gene name could be attached. Plots for coverage versus GC (Blobplots) can be found in Supplementary Fig. S1, and Hi-C contact maps for the assemblies of the four *Diamesa* species can be found in Supplementary Fig. S2.

### Heterozygosity

There was a notable difference in the estimated heterozygosity among the four species sequenced, with *D. hyperborea* and *D. tonsa* (Fig. 1) showing higher levels than *D. lindrothi* and *D. serratosioi* (Table 2, Fig. 3). While more in-depth analyses, preferably with a broader population sampling, would be required to investigate the mechanisms behind this pattern in detail, it aligns with our hypothesis that there is previously occurring or even ongoing introgression between *D. hyperborea* and *D. tonsa*. These two species fly in overlapping time periods at the sample locality in the Rondane Mountains ([73], own observation) and share identical DNA barcodes [30] while harboring considerable differences in their nuclear genomes (see below). A history of interspecific hybridization can explain this pattern.

### Phylogeny

**Figure 4:**
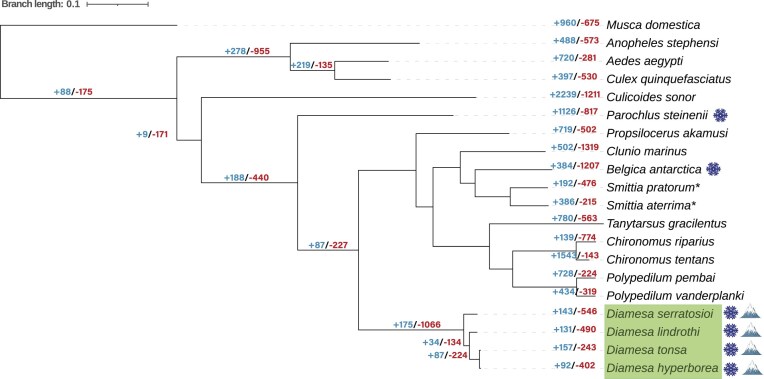
Phylogenomic tree from RAxML and OrthoFinder using single-copy orthogroups rooted with *Musca domestica*. The scale bar represents branch lengths. *Diamesa* are labeled in the green box. A snowflake symbol appears next to species adapted to the cold, while the mountain symbol represents high-altitude adapted species. CAFE analysis was used to determine the number of gene family expansions (in blue) and contractions (in red) for each species and node. *Comparison of COI barcodes indicates that these genomes belong to other species in the genus *Smittia*.

The ML phylogeny based on single-copy orthogroups supports a monophyletic genus *Diamesa* placed as a sister group to the remaining Chironomidae, except *Parochlus* (Fig. 4). This position, as well as the relationship between other genera and subfamilies in our tree, agrees with Cranston et al.’s 4-gene phylogeny that had a considerably broader taxon sampling [74]. It is also consistent with the phylogenomic tree presented by Nell et al. [19].

### Comparative genomics

**Figure 5:**
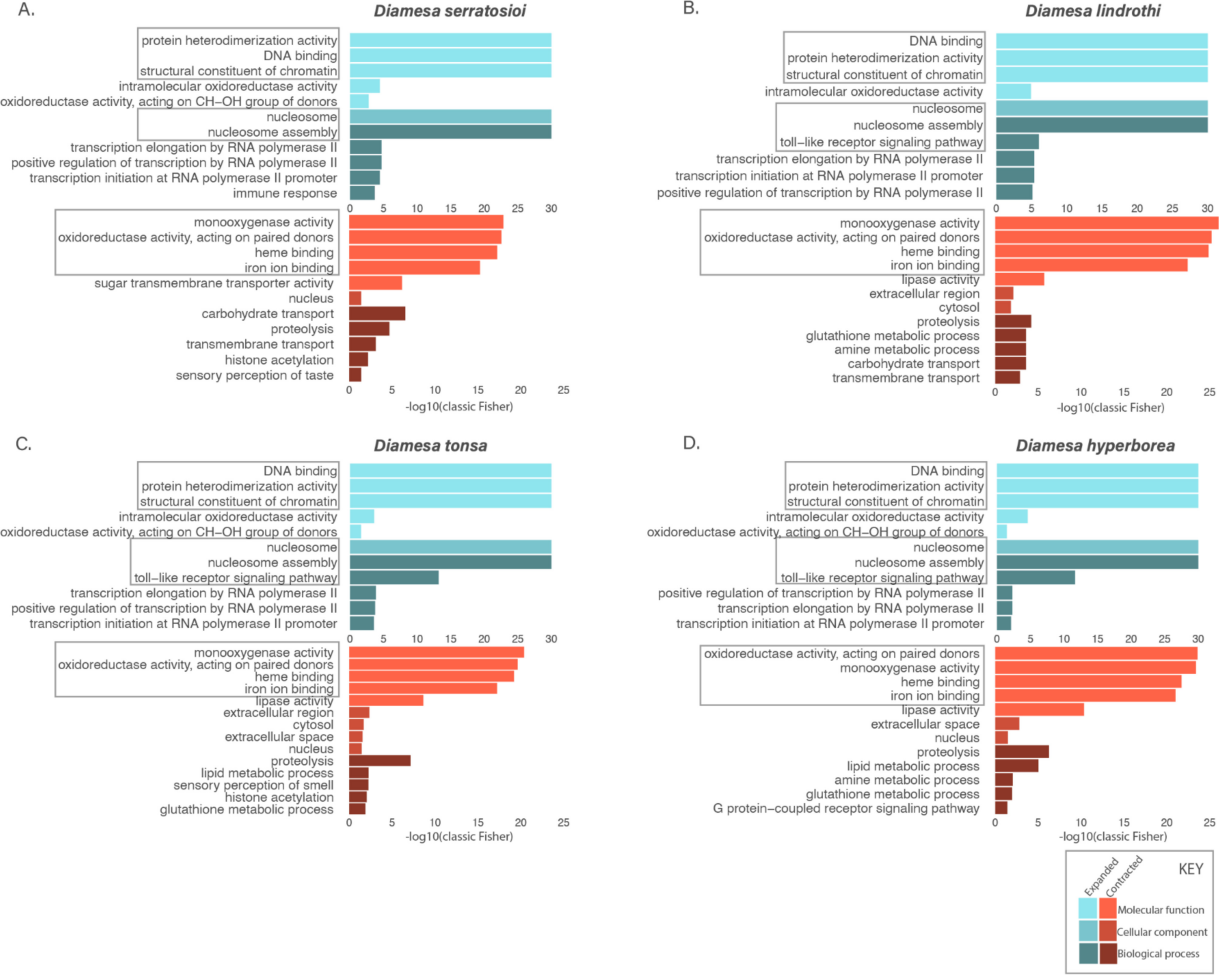
GO analysis of expanded (blue) and contracted (red) gene families. (A) *D. serratosioi*, (B) *D. lindrothi*, (C) *D. tonsa*, and (D) *D. hyperborea*. A maximum of the top five GO terms per class (Biological Process, Cellular Component, and molecular function) are presented. The x-axis is plotted as −log10 (classic Fisher *P* value).

Comparative genomic analysis was used to reveal gene family expansions and contractions within 20 Diptera species’ genomes, with the aim of revealing adaptive divergence of the four *Diamesa* species in response to Arctic conditions, namely, cold temperatures and high altitudes. CAFE [69] analysis resulted in information about the gene expansions and contractions found for each species and each node (Fig. 4). Specifically, we compared primarily the node where our *Diamesa* species diverged from other Diptera (Fig. 4). Expanded and contracted gene families significant at the node for *Diamesa* (Fig. 4), divided into tables for each of the four *Diamesa* species, can be found in Supplementary Tables S3–S10. Results from the GO analysis for the contracted and expanded gene family genes for each of the *Diamesa* species can be found in Supplementary Tables S11 and S12, respectively, and are visualized in Fig. 5.

### *D. tonsa* and *D. hyperborea* are unique species

The large number of differentially expanded and contracted gene families among *D. tonsa* and *D. hyperborea* is strong evidence that these species have undergone divergent evolution and can likely be considered unique species, despite having identical DNA barcodes [30]. While not within the scope of this study, with the published genomes of these species, it is now possible to identify regions of divergence between *D. tonsa* and *D. hyperborea* to facilitate more accurate separation of them using molecular markers from the nuclear genome. Such a study should include additional species in the *cinerella*-group such as *D. cinerella* Meigen, 1835, and *D. hamaticornis* Kieffer, 1924, as these have identical or very similar COI DNA barcodes [13].

### Glucose dehydrogenase plays a crucial role in cold-tolerance adaptation

To explore the similarities between the cold-adapted species *B. antarctica, P. steinenii, D. lindrothi, D. serratosioi, D. hyperborea*, and *D. tonsa*, we searched for gene families with shared contractions or expansions within the genomes of these species. Only one gene family (N0.HOG0000187) was significantly expanded, and none were significantly contracted, in the common ancestor of our four *Diamesa* species, as well as *B. antarctica* and *P. steinenii*, while being nonsignificant in all other species (Supplementary Fig. S3). Notably, this gene family was significantly expanded in the cold-adapted species while being completely absent from *P. vanderplanki*, a desert-dwelling, desiccation-tolerant species, potentially further implicating its importance in cold-adapted species. This gene family includes genes associated with glucose dehydrogenase (GDH) (Uniprot P18173) and ecdysone oxidase (EO) (Uniprot Q9VY01). GDH plays a crucial role in cold adaptation by producing nicotinamide adenine dinucleotide phosphate H (NADPH), which is essential for synthesizing cryoprotectants like sorbitol and glycerol and managing oxidative stress. GDH is involved in the pentose phosphate pathway (PPP), providing NADPH for these processes. Increased GDH activity, and thus NADPH production, in cold-hardy insects may help them survive freezing temperatures by stabilizing cellular structures and reducing damage from reactive oxygen species, in line with previous reports in the goldenrod gall fly *Eurosta solidaginis* [75, 76] and the rice leafroller *Cnaphalocrocis medinalis* [77].

### Genome size as an adaptation to cold temperature and high altitude

Given that there was only one gene family with an expansion or contraction shared between the cold-adapted species, it is likely that the *Diamesa* have further evolved their own unique genomic mechanisms for cold adaptation. There were 1,066 gene family contractions compared to only 175 gene family expansions (Fig. 4), representing the largest gene family contraction of any node. Interestingly, the contraction/expansion ratio is similar to the *B. antarctica* branch while showing the opposite trend in *P. steinenii*. Furthermore, the similarities in genome size for *B. antarctica* (99 Mbp [17]) and the four *Diamesa* species (98–113 Mbp, present study) compared to *P. steinenii* (143 Mbp [24]) could support the hypothesis raised in Kelley et al. [17], suggesting a condensed genome was an evolutionary mechanism for cold tolerance. Clearly, though, this indicates it is not the primary evolutionary strategy for overcoming freezing temperatures, as is the case with *P. steinenii* having a substantially larger genome. To investigate similarities between these small genome cold-adapted Diptera species, we investigated shared gene family contractions/expansions between only *B. antarctica* and the four *Diamesa*. Surprisingly, this resulted in no significantly contracted/expanded gene families in common that were unique to these species, indicating that small genome size could be related to a lack of repeats, transposable elements (TEs), and introns rather than to the dynamism of particular gene families. In fact, the correlation between genome size drivers in Chironomidae was further explored by Nell et al. [19], who determined that the small genome sizes in this family was a result of loss of noncoding regions and repeat elements, although *Diamesa* was not included in their analysis. To better understand the evolutionary mechanisms of the small genome size of *Diamesa*, future detailed comparative analysis of all available chironomid genomes and additional Diamesinae species is required to determine genomic characteristics, such as the number of TEs and repeats, as well as intron lengths, intergenic site lengths, and recombination rate across genomes [5, 78, 79].

### Histone and Toll-like receptor protein gene family expansions in *Diamesa*

Given the lack of similarity in shared gene family dynamics between our *Diamesa* species and other cold-tolerant species in this study, we next focused on the gene contractions and expansions unique to the *Diamesa* node. Of the gene families reported in the *Diamesa* node, 88 and 13 were significantly contracted and expanded, respectively. These gene families were further investigated individually and used to determine enriched GO terms from each list (Supplementary Tables S10 and S11).

The expanded gene families were predominantly related to histones and Toll-like receptor (TLR) proteins and, to a lesser extent, with GDH (discussed above) (Supplementary Tables S3–S6). This was further reflected in the GO analysis of the expanded gene families, which was highly enriched for terms involving chromatin/histone/nucleosome (i.e., GO:0,030,527/GO:0,000,786/GO:0,006,334), as well as TLR proteins (GO:0,002,224) (Fig. 5, Supplementary Table S12). The interplay between the highly enriched nucleosome term with the expanded gene families associated with histones can be explained by their roles in epigenetic gene regulation. In insects, posttranslational histone modifications have been linked to the life span and energy utilization of numerous insects, including in *Eurosta solidaginis* and the goldenrod gall moth *Epiblema scudderiana* (Clemens, 1860) under zero-temperature conditions [80], and even in diapausing mosquitoes [81].

The TLR pathway, an ancient regulatory cascade involved in host defense [82], is essential to the innate immune system in insects, combating fungal and bacterial pathogens. Recent research indicates that environmental conditions, such as cold stress, can influence immune function. In *Drosophila*, cold-induced immune activation is hypothesized to compensate for reduced immune efficiency at low temperatures [83]. Furthermore, environmental temperature has been demonstrated to significantly alter immune responses and the energetic costs of immunity in larvae of the yellow mealworm beetle *Tenebrio molitor* Linnaeus, 1758 [84]. Sinclair et al. [85] reviewed tolerance mechanisms in insects to cold and argued that there is a relationship between low temperatures and the immune response in insects. Thus, the expansion of this gene family in our cold-adapted species further supports this hypothesis.

A study by Kim et al. [86] similarly noted expansions in histone-related gene families as well as GO term enrichment for regulation of the TLR signaling pathway in *B. antarctica* and *P. steinenii*, further highlighting the necessity of these mechanisms for cold adaptation. However, in a more recent comparison of chironomid midges, Nell et al. [19] did not indicate an expansion in gene families associated with histones, chromatin, or TLR signaling pathways, indicating that these expansions occurring in *Diamesa* could have evolved concertedly in cold-tolerant Chironomidae lineages. Further investigations into the role of the expanded histone-related gene families could involve gene expression analyses in *Diamesa*, as well as other cold-tolerant Diptera, at varying temperatures to determine if and which genes are having altered gene expression due to these histone modifications.

### Contractions in gene families associated with oxygen transport and metabolism in *Diamesa*

As mentioned above, there were substantially more gene families undergoing contractions than expansions. This preference for *Diamesa* gene families to contract is likely a result of selective pressures due to rapid changes in their environmental conditions. This is in line with previous findings of gene loss, rather than gene function, contributing to the adaptive evolution of a variety of organisms, from yeast [87] and bacteria [88] to mammals [89] and insects [90]. In a recent study of convergent evolution of high-altitude adapted mammals [89], the authors found that the convergence of the gene family contractions in high-altitude species is much greater than that of expansion, with many of these gene families related to hypoxia response. This study of mammals highlights, in conjunction with our findings in insects, a potential cross-kingdom mechanism of high-altitude animal adaptation that warrants further study. To fully explore this hypothesis, comparative genomic studies are needed in a diversity of high-altitude adapted Diptera species.

More specifically, of the gene annotations within the contracted gene families (Supplementary Tables S7–S10), the terms appeared diverse, as opposed to the clear histone signal in the expanded genes (Supplementary Tables S3–S6). However, after performing GO term enrichment analysis (Fig. 5, Supplementary Table S11), we were able to determine that most significantly enriched GO terms, incorporating oxidoreductase activity, monooxygenase activity, heme binding, and iron ion binding, are related to the function of heme-containing proteins, particularly those involved in oxygen transport and metabolism.

High-altitude animals commonly exhibit positive selection and rapid evolution of genes involved in hypoxia response, suggesting that general genetic mechanisms might be utilized to adapt to high-altitude extremes [91, 92]. Furthermore, changes in oxygen levels in an organism’s environment can drive natural selection for or against genes involved in oxygen metabolism. Previous studies on the convergent evolution of different species, including mammals, fungal pathogens, and insects, also indicated that when species face selection pressure such as environment or eating habits, the expansion and contraction of gene families are mainly the result of direction changes [91, 93]. As such, the contraction of gene families associated with oxygen transport and metabolism could be interpreted as these processes being specialized within these northern *Diamesa*, allowing for a reduction of a larger array of genes controlling these functions, or could indicate less demand of oxygen metabolism in response to their environmental needs (i.e., high altitude and cold temperatures).

While there are no studies reporting contractions of gene families in oxygen transport/metabolism in high-altitude Diptera, a striking overlap between GO terms downregulated in cold-acclimated *D. melanogaster* [94] and *Diamesa* contracted gene families was noted (Fig. 5). This included GO terms associated with oxidoreductase activity (GO:0,016,705), heme binding (GO:0,020,037), iron ion binding (GO:0,005,506), lipase activity (GO:0,016,298), extracellular space (GO:0,005,615) and extracellular region (GO:0,005,576), proteolysis (GO:0,006,508), and lipid metabolic processes (GO:0,006,629). While *Drosophila* and *Diamesa* are not closely related, they both belong to the order Diptera, indicating that these oxygen metabolism processes were already under selection in the *Drosophila* lineage’s response to cold and further evolved as gene redundancies in *Diamesa*.

## Conclusions

This comparative genomic study reveals significant insights into the adaptive mechanisms of *Diamesa* species. The marked contraction of gene families involved in oxygen transport and metabolism suggests a potential reduction in reliance on certain hypoxia-related processes, possibly reflecting adaptations to high-altitude and cold environments where oxygen demand may be decreased or where oxygen metabolism processes are highly specialized. Conversely, the expansion of gene families related to histones and TLR signaling pathways indicates a strategic enhancement of gene regulation and immune responses, likely crucial for survival under extreme cold stress. The notable expansion of glucose dehydrogenase underscores its role in cold tolerance through cryoprotectant synthesis and oxidative stress management. Collectively, these genomic features highlight the complex interplay of gene family contraction and expansion that underpin the unique cold adaptation strategies of *Diamesa* species. Further functional and gene expression studies could elucidate these mechanisms and confirm their roles in ecological resilience to the harsh high-latitude environments.

## Potential Implications

The generation of four haplotype-resolved reference genomes for these nonmodel *Diamesa* species represents a valuable resource for future research. Such high-quality, phased genomes provide a more accurate representation of genetic diversity and facilitate detailed analyses of adaptive variation, gene flow, and evolutionary history. They are essential for identifying structural variants, understanding heterozygosity, and elucidating the genetic basis of traits associated with extreme environments. This genomic resource thus lays a foundation for ongoing studies in ecological genomics, conservation, and evolutionary biology of Arctic and high-altitude insects.

## Additional Files

**Supplementary Fig. S1**. Coverage vs. GC plots of the 4 *Diamesa* species. The BlobToolKit Blobplots depicts each scaffold as a dot based on the GC content (%GC, x-axis) and coverage (y-axis). Size of the dots corresponds to scaffold length. Dots are colored based on assigned taxonomy. Histograms of sequence lengths within a certain %GC range or coverage range are depicted on the top and right, respectively.

**Supplementary Fig. S2**. Hi-C contact map for the assemblies of the 4 *Diamesa* species. The contact map displays interaction frequencies between genomic regions, where darker shades represent a higher number of Hi-C contacts. The axes correspond to the coordinates along each assembly. Hi-C contact maps were generated using PretextMap and visualized using PretextSnapshot.

**Supplementary Fig. S3**. Tree output from OrthoFinder showing the number of significant expansions (red) and contractions (blue) for the gene family N0.HOG0000187, which includes glucose dehydrogenase. The green box indicates the *Diamesa* species, and the yellow boxes indicate the nodes/branches with significant expansions of this gene family. The snowflakes indicate the cold-adapted species. *P < 0.05, **P < 0.01, and ***P < 0.001.

**Supplementary Table S1**. Metadata for *Diamesa* samples collected for high-molecular-weight whole-genome sequencing (wgs) and Hi-C.

**Supplementary Table S2**. Bioinformatic software, versions and sources.

**Supplementary Table S3**. *Diamesa lindrothi* expanded gene families at the *Diamesa* node.

**Supplementary Table S4**. *Diamesa hyperborea* expanded gene families at the *Diamesa* node.

**Supplementary Table S5**. *Diamesa tonsa* expanded gene families at the *Diamesa* node.

**Supplementary Table S6**. *Diamesa serratosioi* expanded gene families at the *Diamesa* node.

**Supplementary Table S7**. *Diamesa lindrothi* contracted gene families at the *Diamesa* node.

**Supplementary Table S8**. *Diamesa hyperborea* contracted gene families at the *Diamesa* node.

**Supplementary Table S9**. *Diamesa tonsa* contracted gene families at the *Diamesa* node.

**Supplementary Table S10**. *Diamesa serratosioi* contracted gene families at the *Diamesa* node.

**Supplementary Table S11**. Top 5 significant GO term for the genes within contracted gene families for the *Diamesa* node.

**Supplementary Table S12**. Top 5 significant GO term for the genes within expanded gene families for the *Diamesa* node.

## Abbreviations

BOLD: Barcode of Life Data Systems; BUSCO: Benchmarking Universal Single-Copy Orthologs; COI: cytochrome c oxidase subunit I; EO: ecdysone oxidase; GDH: glucose dehydrogenase; gDNA: genomic DNA; GO: Gene Ontology; HMW: high molecular weight; HOG: hierarchical orthogroup; ML: maximum likelihood; NADPH: nicotinamide adenine dinucleotide phosphate H; NSC: Norwegian Sequencing Centre; PPP: pentose phosphate pathway; QV: quality value; TLR: Toll-like receptor.

## Ethics Statement

The collection of chironomids within the borders of Rondane National Park was approved by the County Governour of Innlandet and Rondane-Dovre Nasjonalparkstyre (022/5001-4 432.3). Ethics approval was not required for research involving insects. Only the minimum number of individuals was collected to achieve scientific goals.

## Supplementary Material

giaf160_Supplemental_Files

giaf160_Authors_Response_To_Reviewer_Comments_original_submission

giaf160_GIGA-D-25-00303_original_submission

giaf160_GIGA-D-25-00303_Revision_1

giaf160_Reviewer_1_Report_original_submission shijun xiao -- 9/7/2025

giaf160_Reviewer_1_Report_revision_1 shijun xiao -- 11/4/2025

giaf160_Reviewer_2_Report_original_submission Chao Bian, ph.D -- 9/12/2025

giaf160_Reviewer_2_Report_revision_1 Chao Bian, ph.D -- 10/27/2025

## Data Availability

The raw sequencing data for the 4 *Diamesa* species are deposited in the European Nucleotide Archive under accession numbers PRJEB65317, PRJEB96507, PRJEB94282, PRJEB96362, and PRJEB96360. Assembled and annotated genomes, along with protein files, are available on Zenodo [95]. All additional supporting data can be accessed via the *GigaScience* repository, GigaDB [96].
