# Supplementary material for: Haplotype-resolved chromosome-level genome assemblies of four Diamesa species reveal the genetic basis of cold tolerance and high-altitude adaptations in arctic chironomids
Source: Gigascience. 2025 Dec 22;15:giaf160. doi: 10.1093/gigascience/giaf160 (PMC12908713; doi:10.1093/gigascience/giaf160)
Supplement: giaf160_Supplemental_Files [file giaf160_supplemental_files.zip › Diamesa genomes paper Supplementary Figures R1.docx]

**TITLE**

Haplotype-resolved chromosome-level genome assemblies of four *Diamesa* species reveal the genetic basis of cold tolerance and high-altitude adaptations in arctic chironomids

**AUTHORS & AFFILIATIONS**

Sarah L.F. Martin^1*^, Renato La Torre^1^, Bram Danneels^2^, Ave Tooming-Klunderud^3^, Morten Skage^3^, Spyridon Kollias^3^, Ole Kristian Tørresen^3^, Mohsen Falahati Anbaran^1^, Elisabeth Stur^1^, Kjetill S. Jakobsen^3,^ Michael D. Martin^1#^, Torbjørn Ekrem^1#*^

^1^Department of Natural History, NTNU University Museum, Norwegian University for Science and Technology, NO-7491 Trondheim, Norway

^2^Computational Biology Unit, Department of Informatics, University of Bergen, Norway

^3^Centre for Ecological and Evolutionary Synthesis, Department of Biosciences, University of

Oslo, Norway

^#^Indicates shared senior authorship

*Correspondence to: [sarah.martin@ntnu.no](mailto:sarah.martin@ntnu.no), [torbjorn.ekrem@ntnu.no](mailto:torbjorn.ekrem@ntnu.no)


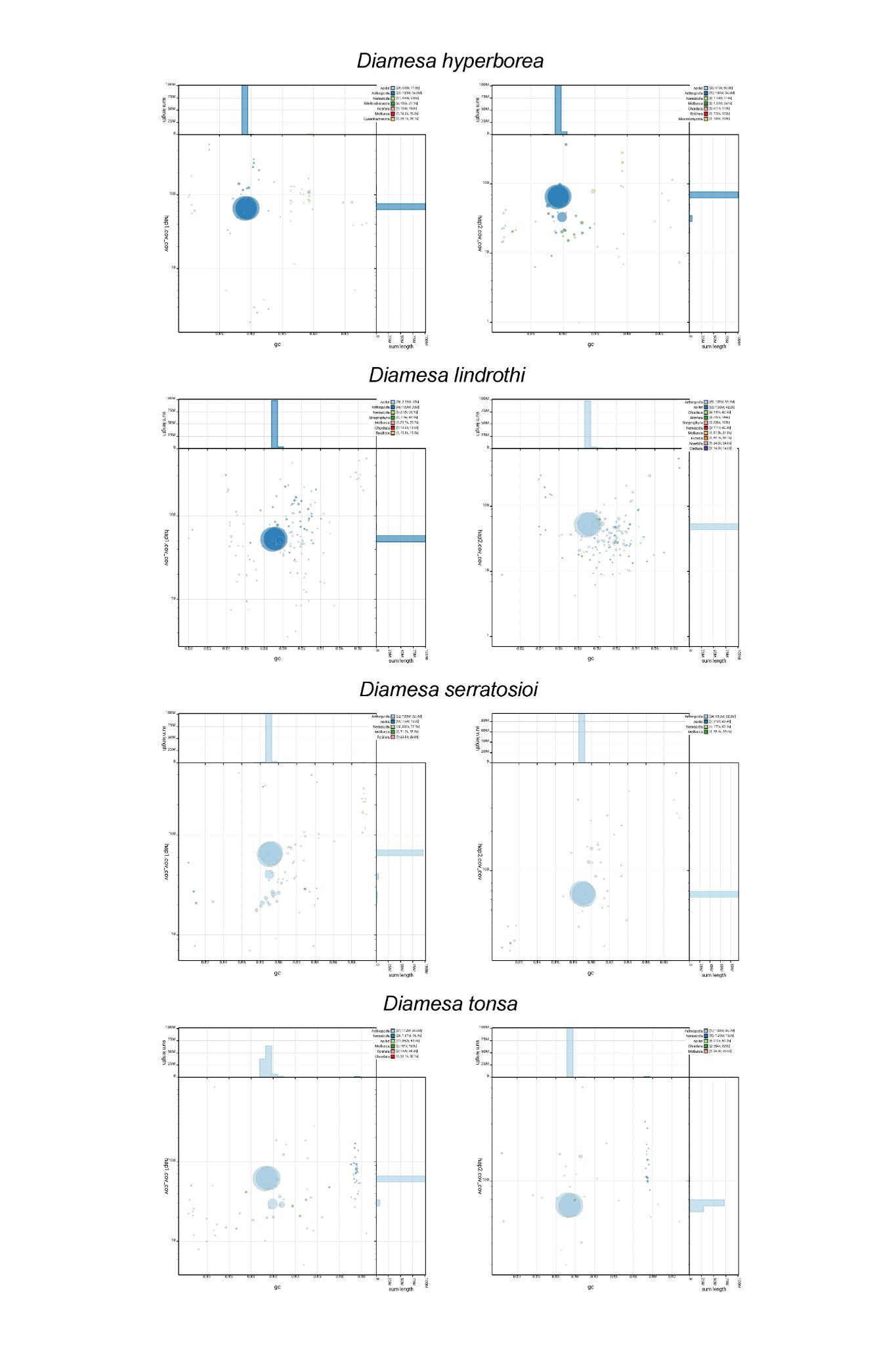
**Supplementary Figure 1: Coverage vs GC plots of the four *Diamesa* species.** The BlobToolKit Blobplots depicts each scaffold as a dot based on the GC content (%GC, x-axis) and coverage (Y-axis). Size of the dots correspond to scaffold length. Dots are colored based on assigned taxonomy. Histograms of sequence lengths within a certain %GC range or coverage range are depicted on the top and right respectively.​


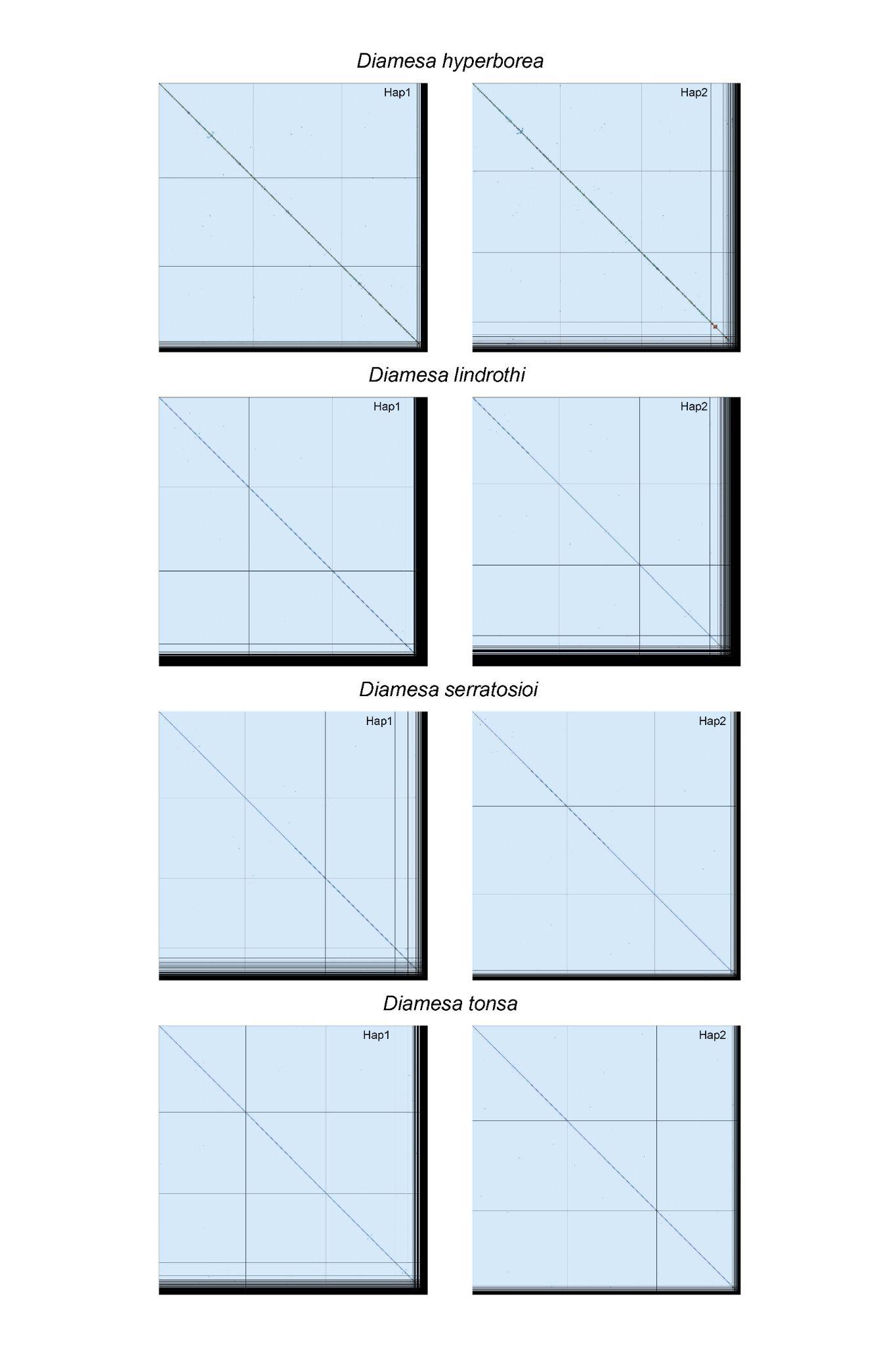


**Supplementary Figure 2**: **Hi-C contact map for the assemblies of the four *Diamesa* species.** The contact map displays interaction frequencies between genomic regions, where darker shades represent a higher number of Hi-C contacts. The axes correspond to the coordinates along each assembly. Hi-C contact maps were generated using PretextMap and visualized using PretextSnapshot.​

**
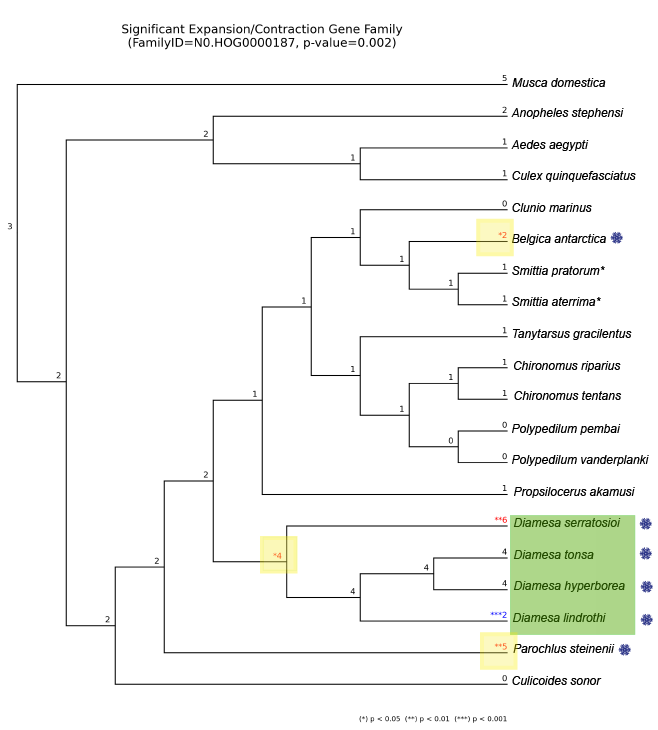
**

**Supplementary Figure 3**: **Tree output from OrthoFinder showing the number of significant expansions (red) and contractions (blue) for the gene family N0.HOG0000187, which includes glucose dehydrogenase**. Significance of p < 0.05 is noted with *, p < 0.01 is noted with **, and p < 0.001 is noted with ***. The green box indicates the *Diamesa* species, the yellow boxes indicate the nodes/branches with significant expansions of this gene family. The snowflakes indicate the cold adapted species.
