# Supplementary material for: Haplotype-resolved chromosome-level genome assemblies of four Diamesa species reveal the genetic basis of cold tolerance and high-altitude adaptations in arctic chironomids
Source: Gigascience. 2025 Dec 22;15:giaf160. doi: 10.1093/gigascience/giaf160 (PMC12908713; doi:10.1093/gigascience/giaf160)
Supplement: giaf160_Authors_Response_To_Reviewer_Comments_original_submission [file giaf160_authors_response_to_reviewer_comments_original_submission.pdf]

Author's Response To Reviewer Comments

Reviewer #1: Using the first haplotype resolved, chromosome-level genomes for four species of Diamesa, authors provide a valuable resource for investigating adaptation, speciation, and conservation in cold-specialist insects and they analyzed the genomic reason, including significant gene family contractions and expansions, for their cold environment adaptations. It effectively highlights the ecological importance of Diamesa midges and the novelty of generating haplotype-resolved, chromosome-level genomes, providing a strong rationale for the study. I think the manuscript could be accepted after authors address the following minor issues:

1. The QV values in Table 2 were evaluated using Hi-C data. Could you clarify the rationale for this approach? In general, Hi-C data are not suitable for assessing genome quality; instead, whole-genome short reads are more appropriate for such evaluations. The relatively low QV value of 20 might be due to the use of Hi-C data, as high-quality short-read evaluations typically yield QV values around 30. If short-read data are available, I recommend re-evaluating the genome quality with Merqury using those reads. If short reads are not available, please provide a reasonable justification for the use of Hi-C data, or retain only the QV evaluation based on HiFi read alignments.

R1.1: Thank you for your attention to our manuscript. Regarding the suggestion to re-evaluate the genome assembly quality, we view it as a waste of resources to perform a separate sequencing round of Illumina reads just for getting a QV score. Using HiFi for QV scores might overestimate the actual QV, because that data is used directly in the assembly. As you say, sequencing data that is not used in the assembly is the best for getting a good QV score. Since the actual sequences of the Hi-C data are not used in the assembly, they provide a more independent score than the HiFi reads, but since it contains k-mers that do not actually occur in the genome (due to the Hi-C protocol of ligating DNA from different places in the genome), the score from Hi-C data should actually indicate a lower boundary. The HiFi data would then be an upper boundary. We feel that providing both gives a better impression of the quality of the assemblies than if we were to provide just one (that would either overestimate or underestimate the actual quality).

2. The title of the manuscript mentions that the genome assemblies are at the chromosome level, and the Conclusions section also refers to chromosome numbers. It would be helpful to include the number of chromosomes in Table 2, which would provide a more intuitive representation of chromosome features and highlight differences among the species.

R1.2: Thank you for this recommendation. We have added this information to Table 2.

3. Based on Supplementary Figure 2 and Table 2, it can be observed that the haplotype carrying the fourth scaffold has a slightly larger genome size and more protein-coding genes than the other haplotype, although the difference is not very pronounced. Could the authors clarify whether this is due to a biological feature of Diamesinae species or a consequence of the assembly process?

R1.3: We appreciate your curiosity about this feature of our genome assembly. We note that our previous version of the manuscript acknowledged this phenomenon (and ascribed it to Diamesa biology) in the second paragraph of the Results: "A scaffold of similar size was detected in the three other species, but in each case was not labelled as chromosome due to its presence in only one of the two haplotypes. The presence of four chromosomes is in line with karyotyping in other Diamesinae species [64]."

4. In addition, BUSCO results are only reported as overall completeness, without distinguishing between single-copy and duplicated genes. It would be helpful to provide this information, as it would give a more complete picture of genome quality and potential assembly artifacts.

R1.4: We agree that reporting the full BUSCO results would indeed be more informative. We have added the full BUSCO strings in the relevant table (Table 2).

Reviewer #2: This paper, entitled 'Haplotype-resolved chromosome-level genome assemblies of four Diamesa species reveal the genetic basis of cold tolerance and high-altitude adaptations in arctic chironomids', provided four chromosome-level genomes of Diamesa by using PCABIO HIFI. Phylogenetics and gene families were identified in this study.

However, I strongly suggest authors to show then expansion result by using some figures. Only expansion introduction is too weak.

R2.1: Thank you for your attention to our manuscript. We had some trouble in interpreting this suggestion, given that we already have Figure 4 showing the numbers of expansions and contractions overlaid on the phylogeny, as well as Figure 5 showing the functional analysis (enriched GO terms) of the expanded and contracted gene families. Given that the reviewer has not suggested a specific figure or information lacking, we are unsure what changes we might make in response to this comment.

We also struggled to understand specifically what the reviewer is referring to when they say “only expansion introduction is too weak”. However, after re-reading the glucose dehydrogenase gene expansion section, we realize that there was some unclear writing here, so we have corrected it to say “Only one gene family (N0.HOG0000187) was found to be significantly expanded, and none were significantly contracted, in the common ancestor of our four Diamesa species, as well as B. antarctica and P. steinenii, while being non-significant in all other species (Supplementary Figure 3). Notably, this gene family was significantly expanded in the cold-adapted species while being completely absent from P. vanderplanki, a desert-dwelling, desiccation-tolerant species, potentially further implicating its importance in cold-adapted species.” We hope that this newly clarified text will satisfy the reviewer.

The genome size condensation is also limited and have no sufficient evidence.

R2.2: As discussed in the section Genome size as an adaptation to cold and high-altitude, we compare the genome size to B. antarctica - another cold-adapted Dipteran, and compare the number of contractions/expansions. While these are not definitive, we feel they are worth mentioning. We have adjusted the language to reflect that this is merely an observation and further analysis would be required to draw firmer conclusions.

Some suggestions:
The format of the abstract should be largely revised.

R2.3:Thank you for pointing this out. We have now reformatted the abstract to meet GigaScience Data Note abstract formatting.

Line 35-37, this sentence is too heavy to understanding, split it to be clearer.
R2.4: Thank you for this suggestion. We have re-written the abstract as advised in the point above.

Line 123-126, the cycle for temperature profile can be removed. I think no need to introduce this too detailed.

R2.5: Thank you for this feedback. We would like to be transparent and include all necessary information for future replication of our work. However, we have re-written this sentence to condense it.

Line 147, "Prior" should be 'prior'.

R2.6: We see no issue with the capitalized P in this instance, given that it occurs at the beginning of a sentence.

Please add the "RRID" and version for the used software.

R2.7: We have added the RRIDs of the used software in Supplementary Table 2.

Line 191, 'samtools' should be 'SAMtools'.

R2.8: Thank you for this good observation. We have corrected the text.

The detailed parameters also need to be shown.

R2.9: The detailed parameters can be found in the Github links for each section - Genome Assembly, Genome Annotation and Genome Evaluation. We determined that including this level of information in the main text was unnecessary, given that it can be found in our Github links. Additionally, software versions, sources and RRIDs have been added to Supplementary Table 2.

For the table 2, this table is bit in disorder. Please move the Hifi read covergage, Hic read coverage and Consensus quanlity, Kmer both, heterozygosity to half bottom of this table.

R2.10: Thank you for this feedback. We have made these changes.

Line 327, 337, Align subheadings to the left margin. Do not indent.

R2.11: Done.

Line 338, 'between' revised to 'among'.
R2.12: Done.

I suggested the authors to initial a figure to show the expansion of Glucose dehydrogenase.
R2.13: We have Supplementary Figure 3 showing the gene family which incorporates Glucose dehydrogenase - this figure shows the phylogeny with the species showing significant expansion highlighted. We have added to the figure’s title that this gene family includes glucose dehydrogenase.

Line 359, 1066 should be '1,066'.
R2.14: We have added this correction.

For the data records, where did the sequenced deposited? There was no NCBI project ID in this study.
R2.15: All data depositions have been added to the manuscript under Data Availability.

Editorial Manager®

Cookie Preference Center

We use cookies which are necessary to make our site work. We may also use additional cookies to analyze, improve and personalize our content and your digital experience. For more information, see our Cookie Policy.

You may choose not to allow some types of cookies. However, blocking some types may

impact your experience of our site and the services we are able to offer. See the different category headings below to find out more or change your settings.

Allow all

Manage Consent Preferences

Strictly Necessary Cookies

Always active

These cookies are necessary for the website to function and cannot be switched off in our systems. They are usually only set in response to actions made by you which amount to a request for services, such as setting your privacy preferences, logging in or filling in forms. You can set your browser to block or alert you about these cookies, but some parts of the site will not then work.

Functional Cookies

These cookies enable the website to provide enhanced functionality and personalisation. They may be set by us or by third party providers whose services we have added to our pages. If you do not allow these cookies then some or all of these services may not function properly.

Performance Cookies

These cookies allow us to count visits and traffic sources so we can measure and improve the performance of our site. They help us to know which pages are the most and least popular and see how visitors move around the site.

Targeting Cookies

These cookies may be set through our site by our advertising partners. They may be used by those companies to build a profile of your interests and show you relevant adverts on other sites. If you do not allow these cookies, you will experience less targeted advertising.

Cookie List

Clear

Apply Cancel

Consent Leg.Interest

Confirm my choices

Powered by onetrus
