# Supplementary material for: Haplotype-resolved chromosome-level genome assemblies of four Diamesa species reveal the genetic basis of cold tolerance and high-altitude adaptations in arctic chironomids
Source: Gigascience. 2025 Dec 22;15:giaf160. doi: 10.1093/gigascience/giaf160 (PMC12908713; doi:10.1093/gigascience/giaf160)
Supplement: giaf160_Reviewer_1_Report_original_submission — shijun xiao -- 9/7/2025 [file giaf160_reviewer_1_report_original_submission.pdf]

Reviewer Report

Title: Haplotype-resolved chromosome-level genome assemblies of four Diamesa species reveal the genetic basis of cold tolerance and high-altitude adaptations in arctic chironomids

Version: Original Submission **Date:** 9/7/2025

Reviewer name: shijun xiao

Reviewer Comments to Author:

Using the first haplotype resolved, chromosome-level genomes for four species of Diamesa, authors provide a valuable resource for investigating adaptation, speciation, and conservation in cold-specialist insects and they analyzed the genomic reason, including significant gene family contractions and expansions, for their cold environment adaptations. It effectively highlights the ecological importance of Diamesa midges and the novelty of generating haplotype-resolved, chromosome-level genomes, providing a strong rationale for the study. I think the manuscript could be accepted after authors address the following minor issues:1ï¼ŽThe QV values in Table 2 were evaluated using Hi-C data. Could you clarify the rationale for this approach? In general, Hi-C data are not suitable for assessing genome quality; instead, whole-genome short reads are more appropriate for such evaluations. The relatively low QV value of 20 might be due to the use of Hi-C data, as high-quality short-read evaluations typically yield QV values around 30. If short-read data are available, I recommend re-evaluating the genome quality with Merqury using those reads. If short reads are not available, please provide a reasonable justification for the use of Hi-C data, or retain only the QV evaluation based on HiFi read alignments.2ï¼ŽThe title of the manuscript mentions that the genome assemblies are at the chromosome level, and the Conclusions section also refers to chromosome numbers. It would be helpful to include the number of chromosomes in Table 2, which would provide a more intuitive representation of chromosome features and highlight differences among the species.3ï¼ŽBased on Supplementary Figure 2 and Table 2, it can be observed that the haplotype carrying the fourth scaffold has a slightly larger genome size and more protein-coding genes than the other haplotype, although the difference is not very pronounced. Could the authors clarify whether this is due to a biological feature of Diamesinae species or a consequence of the assembly process?4ï¼ŽIn addition, BUSCO results are only reported as overall completeness, without distinguishing between single-copy and duplicated genes. It would be helpful to provide this information, as it would give a more complete picture of genome quality and potential assembly artifacts.

Level of Interest

Please indicate how interesting you found the manuscript: Choose an item.

Quality of Written English

Please indicate the quality of language in the manuscript: Choose an item.

Declaration of Competing Interests

Please complete a declaration of competing interests, considering the following questions:

- Have you in the past five years received reimbursements, fees, funding, or salary from an organisation that may in any way gain or lose financially from the publication of this manuscript, either now or in the future?
- Do you hold any stocks or shares in an organisation that may in any way gain or lose financially from the publication of this manuscript, either now or in the future?
- Do you hold or are you currently applying for any patents relating to the content of the manuscript?
- Have you received reimbursements, fees, funding, or salary from an organization that holds or has applied for patents relating to the content of the manuscript?
- Do you have any other financial competing interests?
- Do you have any non-financial competing interests in relation to this paper?

If you can answer no to all of the above, write 'I declare that I have no competing interests' below. If your reply is yes to any, please give details below.

I declare that I have no competing interests

I agree to the open peer review policy of the journal. I understand that my name will be included on my report to the authors and, if the manuscript is accepted for publication, my named report including any attachments I upload will be posted on the website along with the authors' responses. I agree for my report to be made available under an Open Access Creative Commons CC-BY license (http://creativecommons.org/licenses/by/4.0/). I understand that any comments which I do not wish to be included in my named report can be included as confidential comments to the editors, which will not be published.

Choose an item.

To further support our reviewers, we have joined with Publons, where you can gain additional credit to further highlight your hard work (see: https://publons.com/journal/530/gigascience). On publication of this paper, your review will be automatically added to Publons, you can then choose whether or not to claim your Publons credit. I understand this statement.

Yes Choose an item.
