# Supplementary material for: Haplotype-resolved chromosome-level genome assemblies of four Diamesa species reveal the genetic basis of cold tolerance and high-altitude adaptations in arctic chironomids
Source: Gigascience. 2025 Dec 22;15:giaf160. doi: 10.1093/gigascience/giaf160 (PMC12908713; doi:10.1093/gigascience/giaf160)
Supplement: giaf160_Reviewer_2_Report_original_submission — Chao Bian, ph.D -- 9/12/2025 [file giaf160_reviewer_2_report_original_submission.pdf]

Reviewer Report

Title: Haplotype-resolved chromosome-level genome assemblies of four Diamesa species reveal the genetic basis of cold tolerance and high-altitude adaptations in arctic chironomids

Version: Original Submission **Date:** 9/12/2025

Reviewer name: Chao Bian, ph.D

Reviewer Comments to Author:

This paper, entitled 'Haplotype-resolved chromosome-level genome assemblies of fourDiamesa species reveal the genetic basis of cold tolerance and high-altitude adaptations in arctic chironomids', provided four chromosome-level genomes of Diamesa by using PCABIO HIFI. Phylogenetics and gene families were identified in this study.However, I strongly suggest authors to show then expansion result by using some figures. Only expansion introduction is too weak.The genome size condensation is also limited and have no sufficient evidence.Some suggestions:The format of the abstract should be largely revised.Line 35-37, this sentence is too heavy to understanding, split it to be clearer.Line 123-126, the cycle for temperature profile can be removed. I think no need to introduce this too detailed.Line 147, "Prior" should be 'prior'.Please add the "RRID" and version for the used software.Line 191, 'samtools' should be 'SAMtools'.The detailed parameters also need to be shown.For the table 2, this table is bit in disorder. Please move the Hifi read covergage, Hic read coverage and Consensus quanlity, Kmer both, heterozygosity to half bottom of this table.Line 327, 337, Align subheadings to the left margin. Do not indent.Line 338, 'between' revised to 'among'.I suggested the authors to initial a figure to show the expansion of Glucose dehydrogenase.Line 359, 1066 should be '1,066'.For the data records, where did the sequenced deposited? There was no NCBI project ID in this study.

Level of Interest

Please indicate how interesting you found the manuscript: Choose an item.

Quality of Written English

Please indicate the quality of language in the manuscript: Choose an item.

Declaration of Competing Interests

Please complete a declaration of competing interests, considering the following questions:

- Have you in the past five years received reimbursements, fees, funding, or salary from an organisation that may in any way gain or lose financially from the publication of this manuscript, either now or in the future?
- Do you hold any stocks or shares in an organisation that may in any way gain or lose financially from the publication of this manuscript, either now or in the future?

- Do you hold or are you currently applying for any patents relating to the content of the manuscript?
- Have you received reimbursements, fees, funding, or salary from an organization that holds or has applied for patents relating to the content of the manuscript?
- Do you have any other financial competing interests?
- Do you have any non-financial competing interests in relation to this paper?

If you can answer no to all of the above, write 'I declare that I have no competing interests' below. If your reply is yes to any, please give details below.

I declare that I have no competing interests

I agree to the open peer review policy of the journal. I understand that my name will be included on my report to the authors and, if the manuscript is accepted for publication, my named report including any attachments I upload will be posted on the website along with the authors' responses. I agree for my report to be made available under an Open Access Creative Commons CC-BY license (http://creativecommons.org/licenses/by/4.0/). I understand that any comments which I do not wish to be included in my named report can be included as confidential comments to the editors, which will not be published.

Choose an item.

To further support our reviewers, we have joined with Publons, where you can gain additional credit to further highlight your hard work (see: https://publons.com/journal/530/gigascience). On publication of this paper, your review will be automatically added to Publons, you can then choose whether or not to claim your Publons credit. I understand this statement.

Yes Choose an item.
